# Challenges and opportunities in controlling and eradicating neglected tropical diseases: Public and private health operators in Somalia 2025

**DOI:** 10.1186/s41182-025-00885-4

**Published:** 2025-12-27

**Authors:** Saadaq Adan Hussein, Marian Muse Osman, Mohamed Mohamoud Hassan, Abdirahman Aden Hussein, Abdinur Hussein Mohamed, Mohamed Farah Yusuf Mohamud, Rage Adem, Mohamed MAli Fuje, Khadar Hussein Mohamud, Ayan Nur Ali, Abdirahman Moallim Ibrahim, Hassan Ahmed Mohamed, AbdulJalil Abdullahi Ali

**Affiliations:** 1 Department of School of Postgraduate Studies, Benadir University, Mogadishu, Somalia; 2 Department of Social and Human Capital Development Pillar, Office of the Prime Minister, Federal Republic, Mogadishu, Somalia; 3 Department of Research and Policy Development, SOR Institute: Somalia Social Research, Mogadishu, Somalia; 4 Department Benadir Institute for Research and Development, Benadir University, Mogadishu, Somalia; 5 Department Research, Somali National Institute of Health, Mogadishu, Somalia; 6 Department Research, Royal Hospital, Mogadishu, Somalia; 7 Department Rector at, Benadir University, Mogadishu, Somalia; 8 Department of CEO, Tayo Institute for Research and Development, Mogadishu, Somalia; 9 Department Director Innovation Hub, Benadir University, Mogadishu, Somalia; 10 Department Executive of Somali Development Research Institute (SODRI), Mogadishu, Somalia; 11 Department of Hemodialysis at Mogadishu, Somali Türkiye Training and Research Hospital, Mogadishu, Somalia; 12 Departments Faculty of Medicine and Surgery, Jazeera University, Mogadishu, Somalia; 13 Departments Health Management Information Systems, Ministry of Health Mogadishu, Mogadishu, Somalia

**Keywords:** Neglected tropical, Public–private, Surveillance, DHIS2/IDSR, Vector; mHealth

## Abstract

**Background:**

Globally, neglected tropical diseases (NTDs) affect > 1.7 billion people and remain a major cause of morbidity in fragile, resource-limited settings. Somalia, the 44th largest country worldwide, is situated in Africa and ranks among the top 20 countries with the highest number of zero-dose children. Protracted conflict, climate shocks, and displacement hinder effective surveillance and service delivery, exacerbating the prevalence of infectious diseases and active outbreaks. We assessed frontline perspectives on barriers and opportunities for neglected tropical diseases (NTDs) control and eradication in Mogadishu.

**Methodology:**

We conducted a mixed-methods, facility-based cross-sectional study (January–June 2025) across seven hospitals (2 public, 5 private). A structured questionnaire captured sociodemographic and perceptions from 535 health workers (specialists, general practitioners, nurses, pharmacists, midwives, laboratory staff). Qualitative data comprised 15 key informant interviews (KIIs) and 6 focus group discussions (FGDs; 60 participants) with clinicians, CHWs, and managers. Quantitative data were analyzed with descriptive statistics and bivariate tests; qualitative data underwent thematic analysis.

**Result:**

Of 535 participants, 341/535 (63.7%) were female most had 1–5 years of experience and bachelor’s degrees. Training on NTDs was moderate (58.1%). Priority barriers were lack of funding (61.5%), limited infrastructure (17.4%) and low public awareness (14.2%). Perceived opportunities included improved infrastructure (38.5%; *n* = 206), health education (37.8%) vaccination campaigns (32.7%) joint CHW–provider training (46.0%), and mHealth for surveillance/reporting (32.9%) PPPs were viewed as essential/important (89.9%). KIIs/FGDs triangulated these findings, adding themes of stigma (especially leprosy), access barriers in IDP/conflict-affected areas, and underused PPP channels.

**Conclusion:**

NTD progress in Somalia is constrained by financing, capacity, and coordination gaps, but integrated, feasible interventions exist: multi-year financing tied to performance; competency-based upskilling for clinicians/CHWs; standardized CHW–facility referral feedback and digitized reporting within IDSR/DHIS2; formalized PPPs for commodities/reporting/supervision; and climate-aware vector control with WASH messaging prioritized for IDP-dense, flood-prone districts.

## Background

NTDs are a serious threat to people living in tropical and subtropical areas, with severe and potentially fatal consequences [1, 2]. Globally, neglected tropical diseases (NTDs) impact on the lives of more than 1.7 billion individuals [3]. Annually, 200,000 deaths and 19 million exists of life are lost due to disability [4], and 80% of the NTD burden requires treatment for 1.65 billion people [5]. NTD are strongly linked to zoonotic and vector-borne diseases and have significant associations with humans, animals, and the environment [6]. Climate change has increased the prevalence of vector-borne and zoonotic diseases [8]. The current trends in globalization, trade, migration, and international financial markets have a significant impact on the rise, emergence, and re-emergence of transmissible diseases [9]. The WHO Eastern Mediterranean regional office has established programs to prevent and control diseases via the Integrated Disease Surveillance System [9], which aims to detect and respond to all health conditions quickly [10, 11] and Integrated Disease Surveillance and Response improves local response to public health hazards [12].

Africa's sub-Saharan region has the highest concentration of poverty worldwide [13, 14]. The prevalence of NTDs is high among impoverished people in sub-Saharan Africa [15, 16] and most marginalized globally are primarily affected by NTDs [17]. Half of the population of sub-Saharan Africa lives on less than USD 1.25 per day [18] and Africans make $1.9 per day, which is below the poverty line [13]. African countries, particularly Nigeria, DR Congo, Angola, Ethiopia, Ivory Coast, Tanzania, Ghana, Mozambique, South Africa, Sudan, Kenya, Niger, Mali, Cameroon, Central African Republic, Guinea, Congo, and Benin, are most severely affected [20]. There are a lot of campaigns to improve general health and prevent diseases that are strongly connected to this illness [21] and WHO's roadmap for 2021–2030 aims to prevent, control, eliminate, or eradicate 20 NTDs via strategies like mass drug administration and improved sanitation [3, 22].

Somalia there are various exposures and threats, including the possibility of disease outbreaks, and potential health hazards [23, 24]. The health system in Somalia has suffered from decades of civil war [25, 26], climate change-related natural disasters, including drought, poor rains, decreased livestock production, persistent food crises, and, eventually, famines [27, 28] and the worst drought and famine ever since 2022 [28, 29], that led of the weak and worst health indices [30]. Somalia's health security has seen significant progress since 2016, but still faces challenges due to the failure of the Early Warning Alert and Response Network in 2008 [31–33]. Somalia is expected to continue challenges with communicable diseases until 2030 [34]. Zero-dose hotspots are primarily found in rural, nomadic, and conflict-affected areas, with a peak prevalence of 62% [35]. Despite the opportunity Somalia's National Institutes of Health NIH, African Field Epidemiology Network AFENET, Centers for Disease Control and Prevention, United States CDC US and World Health Organization WHO has initiated the Frontline-Field Epidemiology Training Programme (FETP-Frontline) to enhance local disease surveillance and response skills amid political instability [36]. Over two million Somalis have received medication for neglected tropical [37], and still receiving supplies [38]. Although it is a crucial topic, there has not yet been a study conducted in Somalia. This study aims to evaluate challenges and opportunities in controlling NTD in Somalia, bridging the knowledge gap and contributing to understanding the problem.

## Method

### Study setting

The study assessed health professionals in selected hospitals in Mogadishu. The survey was conducted in seven hospitals, including two public and five private hospitals, and carried out various activities of health care. Somalia, located in Africa and bordered by the Gulf of Aden, Djibouti, Ethiopia, Kenya, and the Indian Ocean, is the 44th largest country globally by land area. It has faced significant challenges including conflict, displacement, and climate change, contributing to a public health crisis exacerbated by a high prevalence of infectious diseases and active outbreaks. Notably, Somalia ranks among the top 20 countries for the highest number of zero-dose children, indicating limited access to essential vaccinations. Following the collapse of Siad Barre’s regime in 1991, the country has experienced decades of instability and health challenges. The capital city, Mogadishu, is only the largest city but also serves as its political and economic center, further complicating the health crisis amidst widespread displacement, leaving millions in vulnerable circumstances [26, 35, 39–41]. The public hospital includes De-Martini General Hospital, established in 1946, is a prestigious referral facility in Somalia, offering over 250 beds and a wide range of health services. Benadir Maternity and Children's Hospital, established in 1977, specializes in maternity and childcare services with 700 beds. Shaafi Hospital, established in 2016, is a specialized healthcare facility in Mogadishu, Somalia, with over 100 beds. Dr. Sumait Private Teaching Hospital, managed by SIMAD University, offers comprehensive medical services with different specializations. Aden Abdulle Hospital, a prominent private teaching hospital in Wadajir District, has 14 years of experience and offers specialized care across various departments. Kalkaal Hospital, established in 2015, is a multidisciplinary private healthcare facility in the Hodan District, with over 100 beds. The Somali-Sudan Specialty Hospital, founded in 2014, is a general hospital in Hodon, Mogadishu, Somalia, offering a wide range of multidisciplinary services, focusing on specialty care. With a capacity of over 100 beds, these hospitals are considered valuable assets to the local community in providing medical care.

### Study design

A cross-sectional survey design mixed with quantitative and qualitative was employed, conducted between January and June 2025, to assess the challenges and opportunities related to controlling and eradicating NTDs from the perspective of health professionals working in these facilities. This approach enabled a snapshot of the current state of NTD control efforts, providing valuable insights into the healthcare landscape in Somalia.

### Study population

The study population comprised diverse health professionals from both public and private hospitals. Inclusion criteria required participants to be at least 18 years old, currently working in the selected hospitals, and willing to provide informed consent. The professionals involved in the study were specialists, general practitioners, nurses, pharmacists, midwives, and medical laboratory technicians. Excluded from the study were anesthesiologists, dentists, physiotherapists, optometrists, psychiatrists, nutritionists/dietitians, occupational therapists, and radiographers/radiologic technologists.

### Sample size and sampling method

The study calculated the minimum required sample size using the Cochran formula, assuming a prevalence of 50% and a precision of 5%. After accounting for a 20% non-response rate, the final sample size was determined to be 535 participants,15 key informants (KIs) 6 focus group discussions (FGDs), with a total of 60 participants. A multistage sampling technique was used for participant selection. In the first stage, stratified sampling was employed to categorize the hospitals into public and private sectors. A total of 535 healthcare workers were randomly selected across the two sectors. In the second stage, participants were stratified by their professional groups and selected proportionally based on their numbers. Finally, health workers were consecutively chosen within each professional group until the required sample size was achieved.

### Data collection procedure

Data were collected using a structured, self-administered questionnaire, which was available in both English and Somali to ensure accessibility and comprehension. The questionnaire was adapted from a pre-existing tool designed to assess interventions for NTD. The data collection tool was adapted from [6, 42], a validated source and approved by the Ministry of Health and Human Services' Institutional Review Board, ensures rigor and appropriateness for Somali healthcare facilities. A pilot study was conducted with 35 participants from the target population to test the clarity and relevance of the questions, leading to minor revisions. The final questionnaire contained three sections: sociodemographic information, challenges in NTD control, and opportunities for NTD eradication. Data collection was carried out in-person, with each interview taking approximately 15–20 min. The responses were gathered via Kobo Collect and analyzed with Stata 16 for statistical evaluation. Measures to minimize biases included anonymization and standardized questioning. Future research is recommended to integrate more data sources, such as health facility records and diagnostic information, to enhance the precision of epidemiological findings.

To enhance the understanding of challenges and opportunities in NTD control, qualitative data survey. 15 key informant interviews (KIIs) were conducted with public health experts, policymakers, and healthcare administrators from both public and private hospitals to gather expert insights on systemic issues and policy gaps. Additionally, 6 focus group discussions (FGDs), each with 6 participants (totaling 60 participants), were held with community health workers (CHWs), nurses, and other healthcare professionals. These discussions focused on community-level challenges, including healthcare access, stigma, and environmental factors impacting NTD transmission. These qualitative methods provided contextual depth to the quantitative data, allowing for a comprehensive understanding of NTD control challenges and offering holistic recommendations for healthcare improvements in Somalia.

### Data analysis

Data analysis was conducted using a quantitative approach, with key variables assessed through descriptive and inferential statistics. Descriptive statistics were used to summarize the sociodemographic characteristics of the participants, while the relationship between challenges and opportunities related to NTDs and the sociodemographic factors was analyzed using Chi-square tests and logistic regression. Ratings were based on participants’ responses, categorized as good (7 or more correct answers) and poor (fewer than 7 correct answers). Results were presented in the form of tables and figures to facilitate clear interpretation. The qualitative data were analyzed using thematic analysis with methodical coding in ATLAS.ti to identify recurring themes related to NTD control challenges and opportunities. Codes were assigned to significant patterns, which were then grouped into broader themes to provide a comprehensive understanding of the issues faced.

### Ethical issues

The study received ethical approval from the Institutional Review Board of the Ministry of Health and Human Services Department of Policy and Planning, with approval number Tix: XAG/368/23. Informed consent was obtained from all participants, and privacy and confidentiality were maintained throughout the study. Personal identifying information was not collected, ensuring anonymity in the data. The study adhered to the principles outlined in the Declaration of Helsinki, guaranteeing ethical conduct throughout the research process.

## Results

The study had 535 participants, the majority of hospitals in the sample are private hospitals, accounting for 451 84.3% hospitals, while public hospitals make up the remaining 84 (15.7%) hospitals. the majority being females 194 (36.3) and males 341 (63.7%) of the sample. A total of 535 healthcare professionals participated in the study, with 341 (63.7%) being female. The majority of participants held a bachelor's degree 461 (86.2%), followed by a master's degree 57 (10.7%) and a diploma 17 (3.2%). In terms of professional experience, most healthcare workers had 1–5 years of experience 340 (63.6%), followed by 6–10 years 142 (26.5%). Among the professional roles, nurses made up the largest group (258, 48.2%), followed by general practitioners 133 (24.9%) and specialists 55 (10.3%) (Table 1).

**Table 1:** Socio-demographical characteristics among study participants at selected hospitals

Variable names	Frequency *N* = 535 (%)
Gender	
Male Female	194 (36.3) 341 (63.7)
Education level	
Diploma Bachelor’s degree Master’s degree	17 (3.2) 46 (86.2) 57 (10.7)
Work experience	
< 1 year 1–5 years 6–10 years 11–15 years > 15 years	16 (3) 340 (63.6) 142 (26.5) 29 (5.4) 8 (1.5)
Professional role	
Specialists doctor General practitioner General nursing Pharmacist Midwifery Medical laboratory	55 (10.3) 133 (24.9) 258 (48.2) 33 (6.2) 26 (4.9) 30 (5.6)

The study identified several significant challenges in controlling and eradicating neglected tropical diseases (NTDs) in Somalia. The most commonly cited barrier was lack of funding, reported by 329 (61.5%) participants, followed by inadequate healthcare infrastructure, mentioned by 93 (17.4%). Low public awareness was cited by 76 (14.2%) participants, and poor sanitation and hygiene practices were highlighted by 26 (4.9%). Limited collaboration between public and private health institutions was noted by 7 (1.3%), and lack of access to health services was mentioned by 4 (0.7%). The most common NTDs encountered were leprosy (165, 30.8%), lymphatic filariasis 136 (25.4%), and visceral leishmaniasis 69 (12.9%).

Resources essential for fighting NTDs were found to be lacking. Funding for research and development was the most commonly cited shortfall, with 250 (46.7%) participants reporting this gap. A lack of medical supplies and equipment was noted by 98 (18.3%), and 128 (23.9%) participants reported shortages of trained healthcare personnel. The awareness of NTDs among the general population was reported as moderate by 246 (46.0%), low by 149 (27.9%), and very low by 94 (17.6%), indicating a need for more robust public education programs.

Collaboration with community health workers (CHWs) was also a challenge. Lack of communication and coordination was cited by 255 (47.7%) participants, and 198 (37.0%) reported that inadequate training for CHWs was a significant issue. The level of training received by healthcare professionals on NTDs was moderate for 311 (58.1%) participants (Table 2).

**Table 2:** Challenges for controlling and eradicating neglected tropical infectious diseases

Variable	Frequency *N* = 535 (%)
1. What are the biggest challenges and most significant barriers to effective NTD control and eradication efforts in Somalia?	
A. Lack of funding	329 (61.5)
B. Lack of healthcare infrastructure	93 (17.4)
C. Poor sanitation and hygiene practices	26 (4.9)
D. Lack of access to healthcare services	4 (0.7)
E. Limited collaboration between public and private healthcare institutions	7 (1.3)
F. Lack of public awareness and education	(14.2) 76
2. In your experience, what are the most common neglected tropical infectious diseases encountered in Somalia?	
A. Leprosy	165 (30.8)
B. Lymphatic filariasis-	136 (25.4)
C. Leishmaniosis-visceral leishmaniasis	69 (12.9)
D. Dengue fever	122 (22.8)
E. Chikungunya	19 (3.6)
F. Schistosomiasis	23 (4.3)
G. Soil-transmitted Helminthiases	1 (0.2)
3. What resources are currently lacking in the fight against neglected tropical infectious diseases in Somalia?	
A. Medical supplies and equipment	98 (18.3)
B. Trained healthcare personnel	128 (23.9)
C. Funding for research and development	250 (46.7)
D. Vaccines and treatments	59 (11.0)
4. What is the level of awareness of neglected tropical infectious diseases among the general community/population in Somalia?	
A. High	46 (8.6)
B. Moderate	246 (46.0)
C. Low	149 (27.9)
D. Very low	94 (17.6)
5. What challenges have you faced in collaborating with community health workers to control and eradicate neglected tropical infectious diseases?	
A. Lack of communication and coordination	255 (47.7)
B. Lack of training and education	198 (37.0)
C. Lack of resources	82 (15.3)
6. What is the level of training provided to healthcare professionals on neglected tropical infectious diseases in Somalia?	
A. High	65 (12.1)
B. Moderate	311 (58.1)
C. Low	132 (24.7)
D. Very low	27 (5.0)
7. What challenges have you encountered in providing treatment and care to patients with neglected tropical infectious diseases in Somalia?	
A. Lack of medical supplies and equipment	251 (46.9)
B. Lack of trained healthcare personnel	215 (40.2)
C. Lack of access to healthcare services	65 (12.1)
D. No challenges	4 (0.7)
8. What is the level of collaboration between public and private healthcare institutions in controlling and eradicating neglected tropical infectious diseases in Somalia?	
A. High	218 (40.7)
B. Moderate	242 (45.2)
C. Low	56 (10.5)
D. Very low	19 (3.6)
9. What role do public–private partnerships play in controlling and eradicating neglected tropical infectious diseases in Somalia?	
A. Essential	232 (43.4)
B. Important	249 (46.5)
C. Somewhat important	48 (9)
D. Not important	6 (1.1)
10. What challenges have you encountered in monitoring and evaluating disease control and eradication programs in Somalia?	
A. Lack of data	224 (41.9)
B. Lack of trained personnel	235 (43.9)
C. Lack of resources	76 (14.2)
11. What is the level of support for disease control and eradication efforts from the Somali government?	
A. High	93 (17.4)
B. Moderate	235 (60.7)
C. Low	99 (17.4)

Regarding opportunities to improve NTD control in Somalia, the majority of participants identified increased funding for healthcare 153 (28.6%) and improved healthcare infrastructure 206 (38.5%) as crucial. Health education and awareness programs were seen as important by 202 (37.8%), and vaccination campaigns were supported by 175 (32.7%). Joint training programs between healthcare professionals and CHWs 269 (46.0%) and the use of mobile health technologies for surveillance and reporting 176 (32.9%) were also viewed as critical strategies for improvement (Table 3).

**Table 3:** Opportunities for controlling and eradicating neglected tropical infectious diseases

Variable	Frequency *N* = 535 (%)
1. What opportunities exist for improving disease control and eradication efforts in Somalia?	
A. Increased funding for healthcare	153 (28.6)
B. Improved healthcare infrastructure	206 (38.5)
C. Enhanced collaboration between healthcare professionals and community health workers	115 (21.5)
D. Increased public awareness and education	23 (4.3)
E. All of the above	38 (7.1)
2. What are the most effective approaches for preventing the spread of neglected tropical infectious diseases in Somalia?	
A. Vaccination campaigns	175 (32.7)
B. Health education and awareness programs	202 (37.8)
C. Vector control measures	54 (10.1)
D. Improved sanitation and hygiene practices	19 (3.6)
E. All the above	85 (15.9)
3. What role do community health workers play in controlling and eradicating neglected tropical infectious diseases in Somalia?	
A. Providing healthcare services to underserved communities	222 (41.5)
B. Educating communities about disease prevention and control	162 (30.3)
C. Conducting disease surveillance and reporting	48 (9)
D. All the above	103 (19.3)
4. What strategies have effectively increased public awareness and education regarding neglected tropical infectious diseases in Somalia?	
A. Mass media campaigns	149 (27.9)
B. Community outreach programs	150 (28)
C. Health education in schools	63 (11.8)
D. All of the above	173 (32.3)
5. What opportunities exist for improving collaboration between healthcare professionals and community health workers in controlling and eradicating neglected tropical infectious diseases in Somalia?	
A. Joint training and capacity-building programs	269 (46)
B. Improved communication and coordination	72 (13.5)
C. Sharing of resources and expertise	69 (12.9)
D. All the above	125 (23.4)
6. What opportunities exist for improving the training and education of healthcare professionals on neglected tropical infectious diseases in Somalia?	
A. Increased funding for training programs	89 (16.6)
B. Improved training curricula	175 (32.7)
C. Continuing education programs	93 (17.4)
D. All the above	178 (33.3)
7. What role can international organizations and donor agencies play in supporting Somalia's NTD disease control and eradication efforts?	
A. Providing funding and resources	104 (19.4)
B. Technical assistance and capacity-building	191 (35.7)
C. Advocacy and awareness-raising	76 (12.5)
D. All the above	173 (32.3)
8. What opportunities exist for strengthening disease surveillance and response systems in Somalia?	
A. Improved reporting and data collection	89 (16.6)
B. Enhanced laboratory capacity	60 (11.2)
C. Increased training and resources for disease surveillance teams	193 (36.1)
D. All the above	193 (36.1)
9. What strategies have effectively engaged and mobilized communities to participate in NTD disease control and eradication efforts in Somalia?	
A. Community involvement in program planning and implementation	236 (44.1)
B. Incentives and rewards for participation	92 (17.2)
C. Use of local media and cultural practices	120 (22.4)
D. All the above	87 (16.3)
10. What opportunities exist for leveraging digital health technologies to improve Somalia's NTD disease control and eradication efforts?	
A. Use of mobile health technologies for disease surveillance and reporting	176 (32.9)
B. Telemedicine and remote consultations	94 (17.6)
C. Health information systems for data management and analysis	105 (19.6)
D. All the above	160 (29.9)
11. What role can traditional healers and alternative medicine practitioners play in supporting NTD disease control and eradication efforts in Somalia?	
A. Providing healthcare services to underserved communities	160 (29.9)
B. Educating communities about disease prevention and control	130 (24.3)
C. Collaborating with modern healthcare providers to integrate traditional practices	121 (22.6)
D. All the above	124 (23.2)

Public–private partnerships (PPPs) were seen as essential for NTD control by 232 (43.4%) participants and important by 249 (46.5%). However, their potential was seen as underutilized, with 6 (1.1%) participants considering them not important. The level of support for disease control and eradication efforts by the Somali government was reported as moderate by 235 (60.7%) participants (Table 4).

**Table 4:** Key informant interviews (KIIs)

Theme	Key insights/findings
Funding and resource allocation	NTD programs are heavily reliant on donor funding, limiting sustainability. Local funding is scarce, hindering program scale and reach
Healthcare infrastructure	Rural and conflict-affected areas face significant healthcare challenges, including inadequate facilities and equipment to treat and diagnose NTDs
Public awareness and education	Public education is insufficient, leading to delays in treatment and misconceptions about NTD transmission. Cultural stigma, especially for diseases like leprosy, inhibits seeking help
Public–private partnerships	Limited collaboration between public and private sectors hinders NTD control. The private sector’s potential role remains underutilized
Access to treatment and services	Health services are often inaccessible due to insecurity, geographical barriers, and financial constraints
Government policies and support	Weak enforcement of health regulations and lack of a comprehensive national strategy impede NTD control efforts
Community involvement and empowerment	Empowering local communities and engaging community health workers (CHWs) is essential, but CHWs need more training and support
Environmental and social factors	Climate change and poor sanitation exacerbate NTD spread, particularly in IDP camps and areas prone to flooding

The findings from 6 focus group discussions (FGDs) with 60 participants revealed several key issues at the community level. A significant challenge reported was low awareness of NTDs, with many participants holding misconceptions about disease transmission. Stigma, particularly related to leprosy, was also identified as a barrier to timely treatment. Access to healthcare services was another major issue, especially in rural and conflict-affected areas, where participants reported difficulties due to long distances, high costs, and insecurity. Poor healthcare infrastructure, including the lack of trained personnel and diagnostic tools, was highlighted as a key constraint (Table 5).

**Table 5:** Focus group discussions (FGDs)

Theme	Key insights/findings
Community awareness and education	Awareness of NTDs is low, with misconceptions about transmission. Stigma, especially for diseases like leprosy, prevents timely treatment
Access to healthcare services	Access is hindered by geographical isolation, long distances to health centers, and the high cost of treatment
Healthcare infrastructure and resources	Health centers in rural and conflict zones are poorly equipped, with a shortage of trained personnel and diagnostic tools
Government support and policy	Limited government action and a lack of a cohesive national NTD strategy are major barriers to effective control
Public–private partnerships	Collaboration between public and private sectors is weak, but there is recognition of its importance for improving NTD control
Community engagement and empowerment	Community health workers (CHWs) play a crucial role in NTD control, but need better training and more resources
Sanitation and hygiene	Poor sanitation and overcrowding, particularly in IDP camps, contribute significantly to the spread of NTDs
Climate change and environmental factors	Climate change and increased flooding have worsened the spread of vector-borne diseases like malaria and dengue
Treatment challenges	The high cost of treatment and lack of access to medicines in rural areas are key barriers. Stigma delays treatment for diseases like leprosy
Role of international organizations	While international aid helps, inconsistent support and lack of coordination with local services hinder the effectiveness of aid

## Discussion

### Health system governance and policy

This study identifies significant challenges in the governance of NTD control efforts in Somalia, particularly in policy implementation and coordination. The lack of collaboration between public and private health sectors (46.5%) hinders the efficient management of NTDs, a challenge common in other East African and sub-Saharan African countries. Public–private partnerships (PPPs), although considered essential (43.4%), are underutilized. Strengthening these partnerships through formal frameworks could improve resource allocation, increase coverage, and enhance coordination between sectors [43]. The role of government in enforcing health regulations and creating a comprehensive national strategy is crucial for effective NTD control. Weak enforcement and a lack of coordination remain major barriers to NTD eradication in Somalia, and addressing these through robust policy frameworks could help improve NTD management [44]. Strengthening public–private partnerships (PPPs) and improving policy implementation could enhance NTD control, as seen in Ghana, where formalized PPPs have led to better resource mobilization for disease management [45].

### Health workforce

The study found that the majority of healthcare workers in Somalia are women (63.7%), mirroring global trends in sub-Saharan Africa where women make up the majority of healthcare workers. A predominantly female workforce can significantly enhance community engagement and trust, particularly in rural areas, where women are more likely to reach other women and children. Female healthcare workers improve health education, strengthen prevention efforts, and empower women in healthcare roles, a finding consistent with another research. Despite the high participation of women in the healthcare workforce, the moderate level of education among healthcare workers (58.1%) necessitates the use of simplified tools, such as rapid diagnostic tests (RDTs), to manage NTDs effectively. However, evidence suggests that higher education and training improve diagnostic accuracy and treatment outcomes. Therefore, strengthening educational programs and providing advanced training to healthcare workers would enhance NTD management in Somalia [46, 47] like Tanzania, where training programs for healthcare workers focus on basic diagnostic methods to combat NTDs [48].

### Health service delivery

Health service delivery is heavily impacted by insufficient funding (61.5%) and inadequate healthcare infrastructure (17.4%) in Somalia. These challenges are particularly acute in rural and conflict-affected areas, where healthcare services are often inaccessible due to geographical barriers and insecurity. In addition, the lack of trained personnel and diagnostic tools further exacerbates the problem [49]. Public–private collaborations could be key in overcoming these infrastructure challenges by pooling resources, improving the accessibility of health services, and ensuring that NTD treatment reaches underserved populations. Integrating mobile health technologies (32.9%) for surveillance and reporting could also improve the reach and efficiency of healthcare services, especially in remote areas. These findings align with global health strategies where digital technologies have enhanced disease reporting and healthcare delivery in low-resource settings. A similar situation exists in South Sudan, where conflict and limited infrastructure restrict access to essential healthcare services. Public–private collaborations [50] as seen in Rwanda, could help bridge infrastructure gaps and ensure better service delivery, particularly in underserved areas. Mobile health (mHealth) technologies could enhance surveillance [51] as demonstrated in Malawi, where mHealth has improved disease tracking [52].

### Health financing

One of the most significant challenges identified in this study is the lack of funding, which impedes the scale-up of NTD control programs. A shortage of funds for research and development (46.7%) and the high cost of disease management in Somalia are major barriers to effective NTD control. Despite the high burden of NTDs in resource-poor settings, funding remains limited, often dependent on international donors. Programming aimed at eradicating NTDs requires substantial financial resources, and without adequate funding, progress in NTD control will be slow [53]. The importance of securing long-term, sustainable funding for NTD control cannot be overstated. Strategies such as performance-based funding models could help ensure more effective resource allocation and the sustainability of NTD programs [54–56]. Mozambique faces similar challenges, where limited domestic funding and donor dependency restrict NTD program expansion [57].

### Health information systems

A lack of data (41.9%) and trained personnel (43.9%) for monitoring and evaluating disease control efforts significantly hampers effective NTD management in Somalia. Health information systems are essential for tracking disease progression, monitoring the success of interventions, and making informed decisions about treatment and prevention. However, the absence of accurate data and the lack of skilled personnel in disease surveillance limit the effectiveness of NTD control programs. Strengthening surveillance systems and improving data collection are critical to enhance the ability to respond to NTD outbreaks in real time. The use of integrated disease surveillance systems (IDSR), as adopted by the WHO, could improve data collection and analysis, ultimately improving early detection and response to NTD outbreaks [58]. Increased long-term investment in research and treatment, as seen in Nigeria, is essential for sustainable NTD control [59]. Liberia experiences similar issues, where weak data systems limit the effectiveness of health interventions. Strengthening disease surveillance and improving data collection [60] as seen in Zambia, is essential for more effective NTD management [61].

### Community engagement and empowerment

Community health workers (CHWs) are crucial to NTD control in Somalia, yet challenges remain in their coordination and training. A lack of communication and coordination (47.7%) between CHWs and healthcare professionals is a significant barrier to effective NTD control. Improving communication, providing joint training, and ensuring regular supervision could enhance the role of CHWs in NTD prevention and management. Additionally, low public awareness of NTDs (46.0% moderate, 27.9% low) and the stigma associated with diseases like leprosy contribute to delayed treatment and care-seeking behavior. Public health campaigns that focus on educating communities about NTDs and reducing stigma are essential for improving early diagnosis and treatment. Health education programs, especially in rural and underserved areas, could improve public awareness and facilitate better disease prevention and management [62]. Ethiopia has also faced similar issues but has made progress by formalizing CHW roles and improving their training [63]. In Uganda, CHWs have been integral to improving awareness and prevention efforts, demonstrating the importance of investing in community-based healthcare delivery [64].

### Opportunities for improvement

Several opportunities exist to improve NTD control and eradication efforts in Somalia. The majority of participants identified increased funding (28.6%) and improved healthcare infrastructure (38.5%) as key areas for improvement. Health education programs (37.8%) and vaccination campaigns (32.7%) are also viewed as critical strategies for reducing NTD prevalence. Joint training programs between healthcare professionals and CHWs (46.0%) are seen as an effective means of improving coordination and service delivery. Additionally, digital health technologies like mobile health (mHealth) for disease surveillance and reporting (32.9%) present an opportunity to improve monitoring and reporting of NTDs. Strengthening public–private partnerships could provide additional resources and ensure that NTD programs are adequately funded and resourced. These opportunities align with global strategies for NTD control, where infrastructure development, health education, and technological innovation have proven to be effective [65]. Tanzania, where health education and vaccination campaigns have significantly reduced NTD prevalence. The integration of mobile health technologies for disease surveillance [66] strengthened public–private partnerships are also critical opportunities, as seen in Zambia [67].

### Role of international organizations

International organizations and donor agencies play a significant role in supporting NTD control and eradication efforts in Somalia. Participants highlighted the importance of technical assistance and capacity-building provided by these organizations [68]. Strengthening disease surveillance and response systems through international collaboration is essential for effective NTD control. Enhancing training, improving reporting systems, and providing resources for laboratory capacity are key strategies to strengthen the healthcare infrastructure [69, 70]. Moreover, community engagement through local media and cultural practices could increase the effectiveness of NTD control programs in Somalia. Future research should consider including qualitative insights from these groups to gain a more holistic understanding of NTD control challenges at the grassroots level. This study was independently conducted without institutional funding, allowing for unbiased data analysis.

## Limitations

This study is cross-sectional and relies on self-reported perceptions, which may introduce response and social desirability biases; disease frequencies reflect provider perception rather than laboratory-confirmed incidence. In addition, sampling was urban and facility-based (with many private hospitals), limiting generalizability to rural districts and IDP settings across Somalia. While the cross-sectional design of this study provides insights into the challenges of NTD control at a specific time, it lacks the capacity for causal inferences or trend tracking. Longitudinal studies are recommended to understand temporal trends and causal relationships more dynamically. The focus on Mogadishu limits findings' applicability to rural and internally displaced persons (IDP) areas, where NTD prevalence is higher. Future research should include these settings to capture Somalia's epidemiological diversity better.

## Conclusion

This study provides evidence that can inform policy changes in NTD control strategies in Somalia. Key recommendations include strengthening collaboration with community health workers, expanding training programs, and improving access to diagnostic tools. These policy changes could have a significant impact on NTD prevention and treatment efforts in both urban and rural settings. This mixed-methods assessment highlights financing constraints, health-system capacity gaps, and coordination challenges with CHWs as the principal barriers to NTD control in Mogadishu, with moderate training levels, M&E/data limitations, and stigma/low awareness reinforcing service bottlenecks. Opportunities cluster around infrastructure investment, health education, vaccination campaigns, CHW joint training, digital/mHealth reporting, and formalized PPPs, all aligned with integrated IDSR/DHIS2 strengthening. A pragmatic package—predictable multi-year financing, competency-based upskilling, standardized CHW–facility feedback loops, output-based PPPs, and climate-aware vector control with WASH messaging in IDP-dense areas—offers the most feasible path to accelerate progress toward the NTD Road Map 2030 targets in Somalia. This study provides evidence that can inform policy changes in NTD control strategies in Somalia. Key recommendations include strengthening collaboration with community health workers, expanding training programs, and improving access to diagnostic tools. These policy changes could have a significant impact on NTD prevention and treatment efforts in both urban and rural settings.

## Data Availability

De-identified data can be obtained from the corresponding author.

## Abbreviations

NTDs: Neglected Tropical Diseases
WHO: World Health Organization
USD: United States dollar
DR Congo: Democratic Republic of the Congo
HIV/AIDS: Human Immunodeficiency Virus/Acquired Immunodeficiency Syndrome
IDSR: Integrated Disease Surveillance System
NGO: Non-Governmental Organization
UN: United Nations
DSN: Disease Surveillance and Notification
IRB: Institutional Review Board
CHW: Community Health Worker
KII: Key informant interview
FGD: Focus group discussion
PPP: Public–private partnership

## Declarations

### Descriptions for experiments

No experiments involving human or animal subjects were conducted. This study is based on a narrative review of published literature and publicly available data sources.
